# Phytoplankton-Bacteria Interactions 2.0

**DOI:** 10.3390/microorganisms11061536

**Published:** 2023-06-09

**Authors:** Katherina Petrou

**Affiliations:** School of Life Sciences, University of Technology Sydney, Sydney, NSW 2007, Australia; katherina.petrou@uts.edu.au

There are multiple ways in which phytoplankton and bacteria interact, starting from the fundamental symbiotic associations of direct attachment, through intimate interactions within the phytoplankton phycosphere, to random associations within the water column via the exudation and cycling of dissolved organic carbon (DOC) and other chemical compounds. How these interactions influence growth, cell physiology, community composition, or nutrient cycling is a fascinating area of study with many questions yet to be asked and answered. This special issue (second edition) explores phytoplankton-bacterial interactions from within the phycosphere and surrounding environment to gain insight into various exchanges that influence microbial relationships, cell physiology and biogeochemistry.

The phycosphere can be described as the plume of DOC that surrounds an individual algal cell. It forms a concentration gradient of organic molecules released from the algal cell, which have been shown to attract bacteria within proximity. In this issue, Baker et al. [1] isolate 368 bacterial strains from within phycosphere communities and co-culture them with green algae to assess host growth. This comprehensive study showed that bacteria generally have a positive effect on growth, but most notably a stronger effect on algal carrying capacity, pointing to the more dominant mechanism being the production of toxins as by-products or efficient nutrient remineralisation. Interestingly, they did not observe any phylogenetically-conserved traits in bacterial isolates that had an effect on algal growth, supporting the idea that within the phycosphere, host-bacterial interactions are highly specific. This finding compliments the study by Nef et al. [2], which looked into the importance of bacterially-produced vitamin B12 (cobalamin) for phytoplankton growth, in particular haptophytes. In their study, they explored vitamin B12 acquisition by the haptophyte *Tisochrysis lutea* when co-cultured with B12-producing bacteria in the absence of added B12 in the medium. They found that the haptophyte was unable to obtain sufficient B12 from the bacteria in culture, suggesting that the direct sharing of B12 could be species specific and not a trait of this association. However, the haptophyte’s growth recovered when grown with a consortium of bacteria, emphasising the importance of microbial diversity, where advantageous interactions may instead be the product of the complex networks of microbes found in natural communities.

The size variation across marine microbes is striking, ranging from 0.2–2000 µm, and so too is the space between cells in the vast aqueous environment. Therefore, in nature, the influence of phytoplankton or bacteria on its environment is a function of cell density. Additionally, total cell abundance will determine the distance between cells and thereby encounter rates and interactions in the water as well as with a phycosphere. To quantify how cells might influence each other in an aqueous environment, Omar et al. [3] develop a simulation of diffusional interactions using the reactive oxygen species (ROS) H_2_O_2_ as a model compound. Taking into consideration the decay rates and diffusion gradients from the diatom cell, they found that the H_2_O_2_ fell below background rates within a short distance from the cell. From the size of the simulated diffusional sphere around the diatom cell, the authors conclude that in oligotrophic regions of the ocean, where space between cells is vast, cell-cell encounter and exchange is unlikely, but that likelihood increases in more eutrophic environments, bloom scenarios and within phytoplankton colonies.

At the scale of natural ecosystems, phytoplankton and bacteria play key roles in nutrient cycles and biogeochemistry. One well-described nutrient cycle dominated by phytoplankton and bacterial activity is the marine sulfur cycle. The production of dimethylsulfoniopropionate (DMSP) by marine phytoplankton is efficiently demethylated to form MeSH for bacterial growth or cleaved by heterotrophic bacteria into the climatically active aerosol dimethyl-sulfide (DMS). A review by Jackson and Gabric [4] provides an introduction to the marine sulfur cycle and the microbially-mediated processes involved in the production, conversion and regulation of important sulfur compounds. In particular however, they present the topic in the light of potential climate change impacts on these interactions and processes, with a focus on the influence of rapid ocean warming. Complementary to this theme is the paper by Fernandez et al. [5] In tracing the uptake and distribution of DMSP through a natural marine microbial community, their study reveals that while DMSP cycling was active in large, medium and small size fractions of the community, it was the non-DMSP producing taxa such as diatoms and picoeukaryotes that formed significant reservoirs of DMSP in coastal communities. The location of this study is a known climate change hot spot, with some of the fastest rates of ocean warming and shifting phytoplankton communities. As revealed by the work of Fernandez et al. [5], shifts in phytoplankton community composition would alter the balance of DMSP sources and sinks, therefore as waters warm, we could start to see broader implications for marine sulfur cycling in coastal temperate waters.

The success of oceanic microbes in highly dynamic and heterogeneous environments, is largely attributed to their diversity and adaptability. In the Sub-Antarctic Southern Ocean where Fe is often scarce, Fourquez et al. [6] tested how bacteria and phytoplankton respond to different sources of introduced Fe. Using different types of iron sources (new, regenerated or non-amended) there was considerable versatility in the ability for microbes to access and utilise the different iron supplied, all of which resulted in highly efficient Fe recycling in the surface waters. Their study also highlights that bacterially-regenerated Fe favoured haptophyte growth, whereas new-Fe lead to the rapid proliferation of diatoms. Their work illustrates the importance of understanding interactions between these microbial groups for elucidating the intricacies of ocean biogeochemical cycling.

This collection of papers offers new insights into phytoplankton-bacteria interactions at various spatial scales, from within the phycosphere to within an ecosystem. Together, they highlight some of the ways in which these intimate interactions or loose associations can influence growth, physiology and nutrient cycling. By investigating the minutiae of these two fundamentally important groups together, we can contribute fine-scale detail to the big picture of the functional roles that phytoplankton and bacteria play in aquatic food webs and nutrient cycles.
